# Bis[(2-quinol­yl)methane­diol-κ^2^ *N*,*O*](sulfato-κ*O*)copper(II) dihydrate

**DOI:** 10.1107/S1600536808001980

**Published:** 2008-01-23

**Authors:** Nuno D. Martins, Joana A. Silva, Manuela Ramos Silva, Ana Matos Beja, Abilio J. F. N. Sobral

**Affiliations:** a CEMDRX, Physics Department, University of Coimbra, P-3004-516 Coimbra, Portugal; b Chemistry Department, University of Coimbra, P-3004-516 Coimbra, Portugal

## Abstract

In the title compound, [Cu(SO_4_)(C_10_H_9_NO_2_)_2_]·2H_2_O, the Cu^II^ ion is chelated by two (2-quinol­yl)methane­diol ligands and coordinated by a monodentate sulfate ligand in a distorted trigonal–bipyramidal environment, with O atoms occupying the equatorial sites and N atoms in the axial sites. The dihedral angle between the two essentially planar quinoline ring systems is 45.02 (9)°. In the crystal structure, an extensive O—H⋯O hydrogen-bonding network forms layers parallel to the *ab* plane.

## Related literature

For related literature, see: Zurowska *et al.* (2007 ▶); Dobrzynska *et al.* (2005 ▶); Kumar & Gandotra (1980 ▶); Catterick *et al.* (1974 ▶).
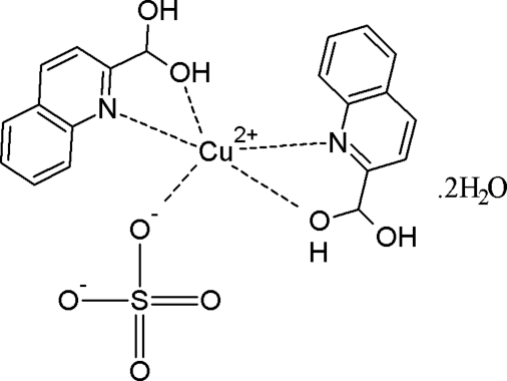


## Experimental

### 

#### Crystal data

[Cu(SO_4_)(C_10_H_9_NO_2_)_2_]·2H_2_O
*M* *_r_* = 546.00
Triclinic, 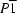

*a* = 7.6065 (3) Å
*b* = 8.8747 (4) Å
*c* = 17.5035 (9) Å
α = 98.561 (3)°
β = 94.324 (3)°
γ = 111.305 (2)°
*V* = 1077.76 (9) Å^3^
*Z* = 2
Mo *K*α radiation
μ = 1.17 mm^−1^
*T* = 293 (2) K
0.24 × 0.15 × 0.12 mm

#### Data collection

Bruker APEX CCD area-detector diffractometer
Absorption correction: multi-scan (*SADABS*; Sheldrick, 2000 ▶) *T*_min_ = 0.810, *T*_max_ = 0.864
18073 measured reflections
5289 independent reflections
3972 reflections with *I* > 2σ(*I*)
*R*_int_ = 0.037

#### Refinement

*R*[*F* ^2^ > 2σ(*F* ^2^)] = 0.037
*wR*(*F* ^2^) = 0.090
*S* = 1.00
5289 reflections
331 parameters
8 restraints
H atoms treated by a mixture of independent and constrained refinement
Δρ_max_ = 0.36 e Å^−3^
Δρ_min_ = −0.34 e Å^−3^

### 

Data collection: *SMART* (Bruker, 2003 ▶); cell refinement: *SAINT* (Bruker, 2003 ▶); data reduction: *SAINT*; program(s) used to solve structure: *SHELXS97* (Sheldrick, 2008 ▶); program(s) used to refine structure: *SHELXL97* (Sheldrick, 2008 ▶); molecular graphics: *ORTEPII* (Johnson, 1976 ▶); software used to prepare material for publication: *SHELXL97*.

## Supplementary Material

Crystal structure: contains datablocks global, I. DOI: 10.1107/S1600536808001980/lh2587sup1.cif

Structure factors: contains datablocks I. DOI: 10.1107/S1600536808001980/lh2587Isup2.hkl

Additional supplementary materials:  crystallographic information; 3D view; checkCIF report

## Figures and Tables

**Table 1:** Selected bond lengths (Å)

Cu1—O5	1.9589 (16)
Cu1—N1	1.9938 (19)
Cu1—N2	1.9969 (19)
Cu1—O3	2.0258 (17)
Cu1—O1	2.1080 (19)

**Table 2:** Hydrogen-bond geometry (Å, °)

*D*—H⋯*A*	*D*—H	H⋯*A*	*D*⋯*A*	*D*—H⋯*A*
O1—H1*A*⋯O6^i^	0.79 (3)	1.77 (3)	2.554 (2)	174 (4)
O3—H3*A*⋯O10	0.83 (3)	1.69 (3)	2.510 (3)	169 (3)
O4—H4*A*⋯O6^ii^	0.83 (3)	2.46 (4)	2.992 (3)	123 (3)
O4—H4*A*⋯O7^ii^	0.83 (3)	2.06 (4)	2.874 (3)	169 (5)
O9—H91⋯O2^iii^	0.86 (4)	2.00 (4)	2.810 (4)	157 (3)
O9—H92⋯O4	0.89 (4)	2.00 (4)	2.879 (5)	178 (6)
O10—H101⋯O9^i^	0.86 (4)	1.91 (4)	2.746 (4)	164 (4)
O10—H102⋯O8^i^	0.84 (3)	2.04 (3)	2.883 (3)	174 (4)

Symmetry codes: (i) 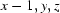; (ii) 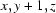; (iii) 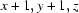.

**Fig. 1.**
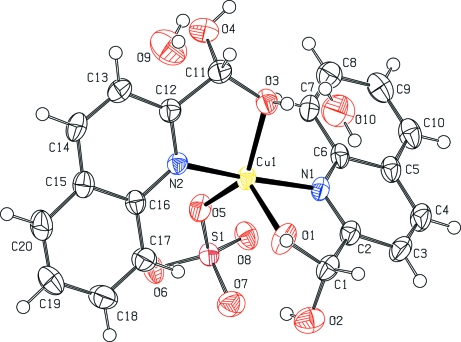
The molecular structure with displacement ellipsoids drawn at the 50% level.

**Fig. 2.**
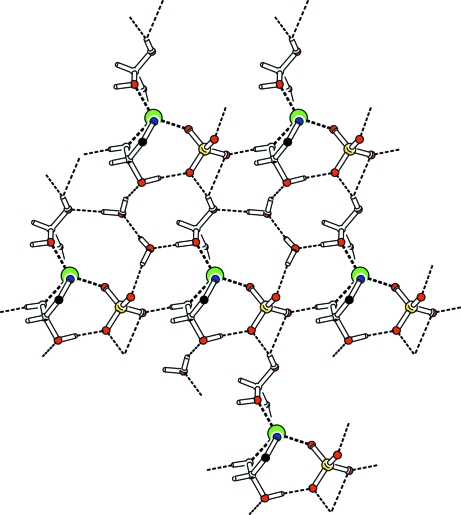
A portion of the H-bond network viewed along the c axis. The hydrohen bonds are depicted as dashed lines.

**Fig. 3.**
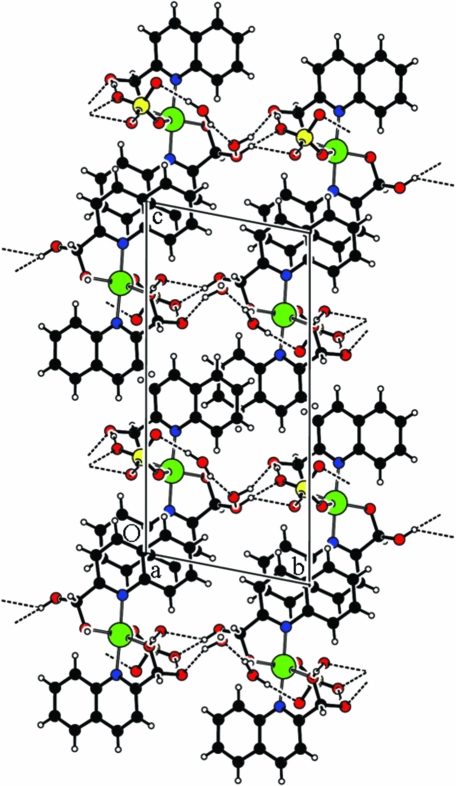
Packing of the title compound, viewed along the a axis. The hydrogen bonds are depicted as dashed lines.

## supplementary crystallographic information

### Comment

Seeking new compounds with low dimensional elements (such as dimers or chains) that may exhibit interesting magnetic properties, we have obtained the title compound, [Cu(C_10_H_9_NO_2_)(SO_4_)].2H_2_O, Fig. 1. There are some examples in literature, where quinoline derivatives ligands were successfully used in the synthesis of compounds with magnetic interactions (Zurowska *et al.*, 2007; Dobrzynska *et al.*, 2005; Kumar & Gandotra, 1980; Catterick *et al.*, 1974). In the title compound, the Cu^II^ ion is surrounded by 5 atoms, 3 oxygen atoms in equatorial positions and two nitrogen atoms in the axial positions delineating a distorted bipyramidal coordination geometry. In the equatorial plane, two of the oxygen atoms are supplied by the hydroxy groups of two symmetry independent quinoline derivatives. The remaining equatorial O atom belongs to a sulfato dinegative ion. The two quinoline derivatives are similar, with the chelating hydroxy groups approximmately sharing the ring plane [O1—C1—C2—N1 - 12.5 (3), O2—C1—C2—N1 109.7 (2)°, and O3—C11—C12—N2 - 1.9 (3), O4—C11—C12—N2 119.0 (2)°]. There is an extensive network of hydrogen bonds forming layers parallel to the *ab* plane (Fig.2 & Fig. 3). The two solvent water molecules are essential in the network formation since they exhaust their capacity of donating and accepting protons. Each pair of water molecules aggregates 3 metal complexes.

.

### Experimental

Approximately 0.13 mmol of 2-quinolinecarboxaldehyde (Sigma, 97%) were dissolved in 2 ml of dichloromethane and then 0.13 mmol of copper sulfate were added to the solution. After one month, single crystals of suitable quality were grown from the solution.

### Refinement

All H-atoms could be located in a Fourier difference map. Aromatic H atoms were positioned geometrically and refined using a riding- model with C—H = 0.93 Å, *U*_iso_(H) = 1.2*U*_eq_(C). Hydroxy and water hydrogen atoms were refined with a distance restraint to their parent O atoms (0.82 and 0.85 Å, respectively), starting from the difference map coordinates and with *U*_iso_(H) = 1.5*U*_eq_(O). There is a short intermolecular contact (H3A ···H101 2.01 Å).

### Crystal data

**Table:**

[Cu(SO_4_)(C_10_H_9_NO_2_)_2_]·2H_2_O	*Z* = 2
*M**_r_* = 546.00	*F*_000_ = 562
Triclinic, *P*1	*D*_x_ = 1.682 Mg m^−^^3^
Hall symbol: -P 1	Mo *K*α radiation λ = 0.71073 Å
*a* = 7.6065 (3) Å	Cell parameters from 4898 reflections
*b* = 8.8747 (4) Å	θ = 2.4–27.5º
*c* = 17.5035 (9) Å	µ = 1.17 mm^−^^1^
α = 98.561 (3)º	*T* = 293 (2) K
β = 94.324 (3)º	Block, green
γ = 111.305 (2)º	0.24 × 0.15 × 0.12 mm
*V* = 1077.76 (9) Å^3^	

### Data collection

**Table:**

Bruker APEX CCD area-detector diffractometer	5289 independent reflections
Radiation source: fine-focus sealed tube	3972 reflections with *I* > 2σ(*I*)
Monochromator: graphite	*R*_int_ = 0.037
*T* = 293(2) K	θ_max_ = 28.4º
φ and ω scans	θ_min_ = 2.4º
Absorption correction: multi-scan(SADABS; Sheldrick, 2000)	*h* = −10→10
*T*_min_ = 0.810, *T*_max_ = 0.864	*k* = −11→11
18073 measured reflections	*l* = −23→22

### Refinement

**Table:**

Refinement on *F*^2^	Secondary atom site location: difference Fourier map
Least-squares matrix: full	Hydrogen site location: inferred from neighbouring sites
*R*[*F*^2^ > 2σ(*F*^2^)] = 0.038	H atoms treated by a mixture of independent and constrained refinement
*wR*(*F*^2^) = 0.090	*w* = 1/[σ^2^(*F*_o_^2^) + (0.0404*P*)^2^ + 0.5543*P*] where *P* = (*F*_o_^2^ + 2*F*_c_^2^)/3
*S* = 1.00	(Δ/σ)_max_ = 0.001
5289 reflections	Δρ_max_ = 0.36 e Å^−^^3^
331 parameters	Δρ_min_ = −0.34 e Å^−^^3^
8 restraints	Extinction correction: none
Primary atom site location: structure-invariant direct methods	

### Special details

**Table:**

Geometry. All e.s.d.'s (except the e.s.d. in the dihedral angle between two l.s. planes) are estimated using the full covariance matrix. The cell e.s.d.'s are taken into account individually in the estimation of e.s.d.'s in distances, angles and torsion angles; correlations between e.s.d.'s in cell parameters are only used when they are defined by crystal symmetry. An approximate (isotropic) treatment of cell e.s.d.'s is used for estimating e.s.d.'s involving l.s. planes.
Refinement. Refinement of *F*^2^ against ALL reflections. The weighted *R*-factor *wR* and goodness of fit *S* are based on *F*^2^, conventional *R*-factors *R* are based on *F*, with *F* set to zero for negative *F*^2^. The threshold expression of *F*^2^ > σ(*F*^2^) is used only for calculating *R*-factors(gt) *etc*. and is not relevant to the choice of reflections for refinement. *R*-factors based on *F*^2^ are statistically about twice as large as those based on *F*, and *R*- factors based on ALL data will be even larger.

### Fractional atomic coordinates and isotropic or equivalent isotropic displacement parameters (Å^2^)

**Table:**

	*x*	*y*	*z*	*U*_iso_*/*U*_eq_	
Cu1	0.34983 (4)	0.15697 (4)	0.247112 (17)	0.02622 (9)	
S1	0.62616 (8)	−0.03258 (7)	0.26884 (3)	0.02588 (14)	
O1	0.0893 (2)	−0.0211 (3)	0.26309 (11)	0.0385 (4)	
H1A	−0.017 (3)	−0.040 (4)	0.2454 (19)	0.058*	
O2	0.1228 (3)	−0.2200 (3)	0.32675 (13)	0.0452 (5)	
H2A	0.233 (3)	−0.201 (4)	0.315 (2)	0.068*	
O3	0.3080 (3)	0.3702 (2)	0.24886 (10)	0.0349 (4)	
H3A	0.198 (3)	0.362 (4)	0.2561 (18)	0.052*	
O4	0.4924 (4)	0.5501 (3)	0.17828 (14)	0.0576 (6)	
H4A	0.499 (6)	0.631 (4)	0.2102 (19)	0.086*	
O5	0.5547 (3)	0.0768 (2)	0.22997 (10)	0.0365 (4)	
O6	0.7384 (2)	−0.0907 (2)	0.21500 (10)	0.0350 (4)	
O7	0.4641 (3)	−0.1762 (2)	0.27989 (12)	0.0427 (5)	
O8	0.7444 (3)	0.0593 (2)	0.34206 (10)	0.0378 (4)	
N1	0.3830 (3)	0.1802 (3)	0.36291 (11)	0.0264 (4)	
N2	0.2914 (3)	0.1435 (2)	0.13256 (11)	0.0262 (4)	
C1	0.0981 (4)	−0.0705 (3)	0.33518 (15)	0.0336 (6)	
H1	−0.0220	−0.0846	0.3558	0.040*	
C2	0.2594 (3)	0.0620 (3)	0.39163 (14)	0.0297 (5)	
C3	0.2741 (4)	0.0530 (4)	0.47104 (16)	0.0374 (6)	
H3	0.1860	−0.0336	0.4888	0.045*	
C4	0.4189 (4)	0.1726 (4)	0.52174 (15)	0.0389 (6)	
H4	0.4306	0.1684	0.5746	0.047*	
C5	0.5511 (4)	0.3028 (3)	0.49378 (14)	0.0316 (6)	
C6	0.5303 (3)	0.3033 (3)	0.41296 (14)	0.0272 (5)	
C7	0.6616 (4)	0.4320 (3)	0.38398 (15)	0.0338 (6)	
H7	0.6501	0.4327	0.3308	0.041*	
C8	0.8053 (4)	0.5555 (4)	0.43358 (17)	0.0399 (6)	
H8	0.8911	0.6401	0.4138	0.048*	
C9	0.8259 (4)	0.5572 (4)	0.51402 (17)	0.0424 (7)	
H9	0.9242	0.6428	0.5472	0.051*	
C10	0.7021 (4)	0.4336 (4)	0.54333 (16)	0.0399 (7)	
H10	0.7168	0.4350	0.5967	0.048*	
C11	0.3207 (4)	0.4235 (3)	0.17647 (15)	0.0356 (6)	
H11	0.2168	0.4609	0.1651	0.043*	
C12	0.3067 (3)	0.2843 (3)	0.11182 (14)	0.0292 (5)	
C13	0.3066 (4)	0.3061 (3)	0.03416 (15)	0.0380 (6)	
H13	0.3209	0.4079	0.0219	0.046*	
C14	0.2855 (4)	0.1769 (4)	−0.02309 (15)	0.0403 (7)	
H14	0.2884	0.1905	−0.0747	0.048*	
C15	0.2594 (4)	0.0225 (3)	−0.00399 (15)	0.0333 (6)	
C16	0.2611 (3)	0.0077 (3)	0.07554 (14)	0.0275 (5)	
C17	0.2298 (4)	−0.1460 (3)	0.09590 (16)	0.0365 (6)	
H17	0.2320	−0.1566	0.1480	0.044*	
C18	0.1964 (4)	−0.2788 (4)	0.03934 (18)	0.0449 (7)	
H18	0.1718	−0.3809	0.0532	0.054*	
C19	0.1977 (4)	−0.2669 (4)	−0.03936 (18)	0.0472 (7)	
H19	0.1777	−0.3595	−0.0769	0.057*	
C20	0.2283 (4)	−0.1192 (4)	−0.06075 (16)	0.0432 (7)	
H20	0.2288	−0.1113	−0.1131	0.052*	
O9	0.8632 (4)	0.5358 (4)	0.20715 (17)	0.0759 (8)	
H91	0.935 (6)	0.628 (4)	0.236 (2)	0.114*	
H92	0.750 (4)	0.541 (6)	0.197 (3)	0.114*	
O10	−0.0100 (3)	0.3450 (3)	0.28886 (17)	0.0614 (6)	
H101	−0.053 (6)	0.390 (5)	0.256 (2)	0.092*	
H102	−0.088 (5)	0.264 (4)	0.304 (2)	0.092*	

### Atomic displacement parameters (Å^2^)

**Table:**

	*U*^11^	*U*^22^	*U*^33^	*U*^12^	*U*^13^	*U*^23^
Cu1	0.03079 (17)	0.03437 (18)	0.01980 (15)	0.01816 (14)	0.00474 (12)	0.00847 (12)
S1	0.0268 (3)	0.0326 (3)	0.0227 (3)	0.0163 (3)	0.0031 (2)	0.0060 (2)
O1	0.0246 (9)	0.0599 (13)	0.0313 (10)	0.0131 (9)	0.0024 (8)	0.0180 (9)
O2	0.0360 (11)	0.0419 (11)	0.0577 (13)	0.0124 (9)	0.0078 (10)	0.0147 (10)
O3	0.0468 (11)	0.0420 (11)	0.0265 (9)	0.0280 (10)	0.0062 (8)	0.0085 (8)
O4	0.0752 (16)	0.0319 (12)	0.0560 (15)	0.0091 (12)	0.0173 (13)	0.0046 (10)
O5	0.0427 (10)	0.0520 (12)	0.0331 (10)	0.0338 (9)	0.0136 (8)	0.0185 (9)
O6	0.0350 (10)	0.0490 (11)	0.0279 (9)	0.0267 (9)	0.0036 (8)	0.0009 (8)
O7	0.0376 (10)	0.0382 (11)	0.0514 (12)	0.0114 (9)	0.0109 (9)	0.0109 (9)
O8	0.0420 (10)	0.0446 (11)	0.0263 (10)	0.0200 (9)	−0.0020 (8)	−0.0007 (8)
N1	0.0295 (10)	0.0361 (12)	0.0212 (10)	0.0189 (9)	0.0077 (8)	0.0098 (9)
N2	0.0284 (10)	0.0299 (11)	0.0232 (10)	0.0138 (9)	0.0033 (8)	0.0073 (9)
C1	0.0284 (13)	0.0435 (16)	0.0345 (14)	0.0155 (12)	0.0105 (11)	0.0164 (12)
C2	0.0320 (13)	0.0385 (14)	0.0273 (13)	0.0203 (12)	0.0089 (11)	0.0118 (11)
C3	0.0447 (16)	0.0439 (16)	0.0328 (15)	0.0210 (13)	0.0145 (13)	0.0204 (13)
C4	0.0539 (17)	0.0504 (17)	0.0229 (13)	0.0291 (15)	0.0084 (12)	0.0133 (13)
C5	0.0387 (14)	0.0424 (15)	0.0229 (12)	0.0255 (13)	0.0052 (11)	0.0071 (11)
C6	0.0290 (12)	0.0372 (14)	0.0220 (12)	0.0201 (11)	0.0063 (10)	0.0052 (11)
C7	0.0369 (14)	0.0427 (15)	0.0261 (13)	0.0182 (12)	0.0105 (11)	0.0085 (12)
C8	0.0355 (14)	0.0413 (16)	0.0426 (16)	0.0140 (13)	0.0085 (13)	0.0078 (13)
C9	0.0399 (16)	0.0475 (17)	0.0391 (16)	0.0216 (14)	−0.0015 (13)	−0.0037 (14)
C10	0.0467 (16)	0.0537 (18)	0.0256 (14)	0.0299 (15)	−0.0012 (12)	0.0018 (13)
C11	0.0486 (16)	0.0365 (15)	0.0318 (14)	0.0248 (13)	0.0079 (12)	0.0135 (12)
C12	0.0319 (13)	0.0347 (14)	0.0264 (13)	0.0176 (11)	0.0042 (10)	0.0088 (11)
C13	0.0501 (16)	0.0392 (15)	0.0312 (14)	0.0204 (13)	0.0085 (12)	0.0160 (12)
C14	0.0501 (17)	0.0518 (18)	0.0219 (13)	0.0197 (14)	0.0080 (12)	0.0135 (13)
C15	0.0326 (13)	0.0415 (15)	0.0243 (13)	0.0133 (12)	0.0022 (10)	0.0045 (11)
C16	0.0272 (12)	0.0334 (13)	0.0225 (12)	0.0135 (11)	0.0013 (10)	0.0034 (10)
C17	0.0467 (16)	0.0333 (14)	0.0299 (14)	0.0162 (13)	0.0008 (12)	0.0066 (12)
C18	0.0548 (18)	0.0315 (15)	0.0453 (18)	0.0158 (14)	0.0011 (14)	0.0028 (13)
C19	0.0548 (19)	0.0419 (17)	0.0369 (16)	0.0170 (15)	−0.0004 (14)	−0.0092 (14)
C20	0.0490 (17)	0.0507 (18)	0.0245 (14)	0.0165 (15)	0.0019 (12)	−0.0013 (13)
O9	0.080 (2)	0.0642 (17)	0.0698 (19)	0.0223 (16)	−0.0045 (16)	−0.0083 (14)
O10	0.0448 (13)	0.0588 (16)	0.0801 (19)	0.0174 (12)	0.0190 (13)	0.0126 (14)

### Geometric parameters (Å, °)

**Table:**

Cu1—O5	1.9589 (16)	C6—C7	1.407 (3)
Cu1—N1	1.9938 (19)	C7—C8	1.361 (4)
Cu1—N2	1.9969 (19)	C7—H7	0.9300
Cu1—O3	2.0258 (17)	C8—C9	1.402 (4)
Cu1—O1	2.1080 (19)	C8—H8	0.9300
S1—O8	1.4486 (19)	C9—C10	1.357 (4)
S1—O7	1.4664 (19)	C9—H9	0.9300
S1—O6	1.4766 (17)	C10—H10	0.9300
S1—O5	1.4914 (17)	C11—C12	1.512 (4)
O1—C1	1.401 (3)	C11—H11	0.9800
O1—H1A	0.79 (2)	C12—C13	1.401 (3)
O2—C1	1.394 (3)	C13—C14	1.358 (4)
O2—H2A	0.84 (2)	C13—H13	0.9300
O3—C11	1.415 (3)	C14—C15	1.407 (4)
O3—H3A	0.83 (2)	C14—H14	0.9300
O4—C11	1.373 (3)	C15—C20	1.414 (4)
O4—H4A	0.83 (2)	C15—C16	1.417 (3)
N1—C2	1.322 (3)	C16—C17	1.402 (3)
N1—C6	1.376 (3)	C17—C18	1.353 (4)
N2—C12	1.320 (3)	C17—H17	0.9300
N2—C16	1.381 (3)	C18—C19	1.398 (4)
C1—C2	1.512 (4)	C18—H18	0.9300
C1—H1	0.9800	C19—C20	1.360 (4)
C2—C3	1.402 (3)	C19—H19	0.9300
C3—C4	1.360 (4)	C20—H20	0.9300
C3—H3	0.9300	O9—H91	0.86 (2)
C4—C5	1.409 (4)	O9—H92	0.89 (2)
C4—H4	0.9300	O10—H101	0.86 (2)
C5—C6	1.412 (3)	O10—H102	0.84 (2)
C5—C10	1.414 (4)		
			
O5—Cu1—N1	96.19 (7)	N1—C6—C5	120.6 (2)
O5—Cu1—N2	90.66 (7)	C7—C6—C5	119.2 (2)
N1—Cu1—N2	173.14 (8)	C8—C7—C6	120.2 (2)
O5—Cu1—O3	137.56 (8)	C8—C7—H7	119.9
N1—Cu1—O3	94.29 (7)	C6—C7—H7	119.9
N2—Cu1—O3	80.56 (7)	C7—C8—C9	121.0 (3)
O5—Cu1—O1	115.59 (8)	C7—C8—H8	119.5
N1—Cu1—O1	79.02 (8)	C9—C8—H8	119.5
N2—Cu1—O1	98.05 (8)	C10—C9—C8	119.9 (3)
O3—Cu1—O1	106.76 (8)	C10—C9—H9	120.1
O8—S1—O7	111.92 (12)	C8—C9—H9	120.1
O8—S1—O6	110.54 (10)	C9—C10—C5	120.9 (3)
O7—S1—O6	108.23 (11)	C9—C10—H10	119.6
O8—S1—O5	109.74 (11)	C5—C10—H10	119.6
O7—S1—O5	109.44 (11)	O4—C11—O3	110.7 (2)
O6—S1—O5	106.82 (10)	O4—C11—C12	107.5 (2)
C1—O1—Cu1	112.67 (15)	O3—C11—C12	110.4 (2)
C1—O1—H1A	111 (2)	O4—C11—H11	109.4
Cu1—O1—H1A	131 (2)	O3—C11—H11	109.4
C1—O2—H2A	106 (3)	C12—C11—H11	109.4
C11—O3—Cu1	113.40 (14)	N2—C12—C13	122.8 (2)
C11—O3—H3A	101 (2)	N2—C12—C11	116.6 (2)
Cu1—O3—H3A	113 (2)	C13—C12—C11	120.6 (2)
C11—O4—H4A	110 (3)	C14—C13—C12	119.4 (2)
S1—O5—Cu1	132.80 (11)	C14—C13—H13	120.3
C2—N1—C6	119.1 (2)	C12—C13—H13	120.3
C2—N1—Cu1	116.29 (17)	C13—C14—C15	119.7 (2)
C6—N1—Cu1	124.51 (16)	C13—C14—H14	120.1
C12—N2—C16	119.3 (2)	C15—C14—H14	120.1
C12—N2—Cu1	114.79 (16)	C14—C15—C20	122.9 (2)
C16—N2—Cu1	125.41 (15)	C14—C15—C16	118.5 (2)
O2—C1—O1	110.8 (2)	C20—C15—C16	118.6 (2)
O2—C1—C2	110.6 (2)	N2—C16—C17	120.2 (2)
O1—C1—C2	109.4 (2)	N2—C16—C15	120.2 (2)
O2—C1—H1	108.7	C17—C16—C15	119.6 (2)
O1—C1—H1	108.7	C18—C17—C16	119.6 (3)
C2—C1—H1	108.7	C18—C17—H17	120.2
N1—C2—C3	123.0 (2)	C16—C17—H17	120.2
N1—C2—C1	117.7 (2)	C17—C18—C19	121.9 (3)
C3—C2—C1	119.2 (2)	C17—C18—H18	119.1
C4—C3—C2	119.2 (3)	C19—C18—H18	119.1
C4—C3—H3	120.4	C20—C19—C18	119.7 (3)
C2—C3—H3	120.4	C20—C19—H19	120.1
C3—C4—C5	119.6 (2)	C18—C19—H19	120.1
C3—C4—H4	120.2	C19—C20—C15	120.5 (3)
C5—C4—H4	120.2	C19—C20—H20	119.7
C4—C5—C6	118.5 (2)	C15—C20—H20	119.7
C4—C5—C10	122.7 (2)	H91—O9—H92	107 (4)
C6—C5—C10	118.8 (2)	H101—O10—H102	118 (4)
N1—C6—C7	120.1 (2)		
			
O5—Cu1—O1—C1	71.43 (18)	C2—N1—C6—C5	0.8 (3)
N1—Cu1—O1—C1	−20.23 (17)	Cu1—N1—C6—C5	−174.93 (16)
N2—Cu1—O1—C1	166.06 (17)	C4—C5—C6—N1	0.7 (3)
O3—Cu1—O1—C1	−111.44 (17)	C10—C5—C6—N1	−178.7 (2)
O5—Cu1—O3—C11	62.2 (2)	C4—C5—C6—C7	−179.7 (2)
N1—Cu1—O3—C11	166.21 (18)	C10—C5—C6—C7	0.9 (3)
N2—Cu1—O3—C11	−18.35 (18)	N1—C6—C7—C8	178.8 (2)
O1—Cu1—O3—C11	−113.98 (18)	C5—C6—C7—C8	−0.8 (3)
O8—S1—O5—Cu1	−78.16 (18)	C6—C7—C8—C9	0.1 (4)
O7—S1—O5—Cu1	45.01 (19)	C7—C8—C9—C10	0.5 (4)
O6—S1—O5—Cu1	161.97 (15)	C8—C9—C10—C5	−0.4 (4)
N1—Cu1—O5—S1	35.82 (18)	C4—C5—C10—C9	−179.7 (2)
N2—Cu1—O5—S1	−144.19 (17)	C6—C5—C10—C9	−0.4 (4)
O3—Cu1—O5—S1	139.14 (15)	Cu1—O3—C11—O4	−103.0 (2)
O1—Cu1—O5—S1	−44.94 (19)	Cu1—O3—C11—C12	16.0 (3)
O5—Cu1—N1—C2	−101.70 (17)	C16—N2—C12—C13	−4.5 (4)
O3—Cu1—N1—C2	119.48 (17)	Cu1—N2—C12—C13	167.7 (2)
O1—Cu1—N1—C2	13.22 (16)	C16—N2—C12—C11	174.3 (2)
O5—Cu1—N1—C6	74.10 (18)	Cu1—N2—C12—C11	−13.5 (3)
O3—Cu1—N1—C6	−64.71 (18)	O4—C11—C12—N2	119.0 (2)
O1—Cu1—N1—C6	−170.97 (18)	O3—C11—C12—N2	−1.9 (3)
O5—Cu1—N2—C12	−120.78 (17)	O4—C11—C12—C13	−62.2 (3)
O3—Cu1—N2—C12	17.49 (17)	O3—C11—C12—C13	176.9 (2)
O1—Cu1—N2—C12	123.25 (17)	N2—C12—C13—C14	1.6 (4)
O5—Cu1—N2—C16	50.87 (19)	C11—C12—C13—C14	−177.1 (3)
O3—Cu1—N2—C16	−170.9 (2)	C12—C13—C14—C15	1.6 (4)
O1—Cu1—N2—C16	−65.10 (19)	C13—C14—C15—C20	177.2 (3)
Cu1—O1—C1—O2	−99.7 (2)	C13—C14—C15—C16	−1.7 (4)
Cu1—O1—C1—C2	22.5 (2)	C12—N2—C16—C17	−175.1 (2)
C6—N1—C2—C3	−1.9 (3)	Cu1—N2—C16—C17	13.6 (3)
Cu1—N1—C2—C3	174.13 (18)	C12—N2—C16—C15	4.2 (3)
C6—N1—C2—C1	179.38 (19)	Cu1—N2—C16—C15	−167.07 (17)
Cu1—N1—C2—C1	−4.6 (3)	C14—C15—C16—N2	−1.2 (4)
O2—C1—C2—N1	109.7 (2)	C20—C15—C16—N2	179.9 (2)
O1—C1—C2—N1	−12.5 (3)	C14—C15—C16—C17	178.1 (2)
O2—C1—C2—C3	−69.0 (3)	C20—C15—C16—C17	−0.8 (4)
O1—C1—C2—C3	168.7 (2)	N2—C16—C17—C18	178.6 (2)
N1—C2—C3—C4	1.6 (4)	C15—C16—C17—C18	−0.7 (4)
C1—C2—C3—C4	−179.8 (2)	C16—C17—C18—C19	2.0 (5)
C2—C3—C4—C5	0.0 (4)	C17—C18—C19—C20	−1.8 (5)
C3—C4—C5—C6	−1.1 (4)	C18—C19—C20—C15	0.2 (5)
C3—C4—C5—C10	178.3 (2)	C14—C15—C20—C19	−177.9 (3)
C2—N1—C6—C7	−178.8 (2)	C16—C15—C20—C19	1.0 (4)
Cu1—N1—C6—C7	5.5 (3)		

### Hydrogen-bond geometry (Å, °)

**Table:**

*D*—H···*A*	*D*—H	H···*A*	*D*···*A*	*D*—H···*A*
O1—H1A···O6^i^	0.79 (3)	1.77 (3)	2.554 (2)	174 (4)
O3—H3A···O10	0.83 (3)	1.69 (3)	2.510 (3)	169 (3)
O4—H4A···O6^ii^	0.83 (3)	2.46 (4)	2.992 (3)	123 (3)
O4—H4A···O7^ii^	0.83 (3)	2.06 (4)	2.874 (3)	169 (5)
O9—H91···O2^iii^	0.86 (4)	2.00 (4)	2.810 (4)	157 (3)
O9—H92···O4	0.89 (4)	2.00 (4)	2.879 (5)	178 (6)
O10—H101···O9^i^	0.86 (4)	1.91 (4)	2.746 (4)	164 (4)
O10—H102···O8^i^	0.84 (3)	2.04 (3)	2.883 (3)	174 (4)

Symmetry codes: (i) *x*−1, *y*, *z*; (ii) *x*, *y*+1, *z*; (iii) *x*+1, *y*+1, *z*.
